# The Financial and Psychological Impact of Identity Theft Among Older Adults

**DOI:** 10.1093/geroni/igab043

**Published:** 2021-10-05

**Authors:** Marguerite DeLiema, David Burnes, Lynn Langton

**Affiliations:** 1 University of Minnesota School of Social Work, Saint Paul, Minnesota, USA; 2 Factor-Inwentash Faculty of Social Work, University of Toronto, Toronto, Ontario, Canada; 3 Applied Justice Research Division, RTI International, Research Triangle Park, North Carolina, USA

**Keywords:** Emotional distress, Fraud, Poverty, Socioeconomic status, Victimization

## Abstract

**Background and Objectives:**

Society’s growing reliance on technology to transfer private information has created more opportunities for identity thieves to access and misuse personal data. Research on identity theft specifically among adults aged 65 and older is virtually nonexistent, yet research focusing on victims of all ages indicates a positive association between age, minority status, and more severe economic and psychological consequences.

**Research Design and Methods:**

Identity theft measures come from a sample of more than 2,000 self-reported victims aged 65 and older from the nationally representative National Crime Victimization Survey Identity Theft Supplements administered in 2014 and 2016. Regression was used to examine how socioeconomic status, demographic characteristics, and incident-specific factors relate to how much money is stolen, the likelihood of experiencing out-of-pocket costs, and emotional distress among older identity theft victims.

**Results:**

Older Black identity theft victims were more likely to have greater amounts of money stolen and were more likely to feel distressed by the incident than older White victims. The most disadvantaged older adults living at or below the federal poverty level were significantly more likely to suffer out-of-pocket costs. The length of time information was misused, experiencing subsequent financial problems and problems with friends/family, and the hours spent resolving identity theft were positively associated with emotional distress. Among those aged 65 and older, age was not significantly associated with losses or emotional distress.

**Discussion and Implications:**

Older adults living in poverty need more resources to assist with recovery and reporting identity theft to law enforcement. Limiting the extent of losses from identity theft and reducing the length of time information is misused may reduce the emotional toll of identity theft on older victims.

**Translational Significance:** More than 7% of older adults are victims of identity theft each year, and a third experience moderate to severe emotional distress following the incident. We find that victims who can least afford it suffer out-of-pocket costs, and that Black and female victims are more likely to report distress. Victim service organizations should pay special attention to these groups and individuals who lack the social capital to advocate for their financial recovery. Greater psychological support is needed to help older adults recover, in addition to training on how to protect their information from future misuse.

There is a growing body of research on the predictors and consequences of financial victimization of older adults. Existing research focuses primarily on two types of victimization—financial abuse/exploitation (a form of elder abuse), in which the perpetrator occupies a position of expected trust like a friend, family member, or caregiver (Hall et al., 2016); and financial fraud and scams, where a stranger uses a false promise or fabricated threat to deceive the victim into paying money (DeLiema, 2018). Limited research to date has examined the impact of the third form of financial victimization—identity theft—on older adults, despite the increasing prevalence of this serious crime (Harrell, 2019).

Identity theft is the intentional, unauthorized use of a person’s identifying information for unlawful purposes (Federal Trade Commission [FTC], 1998). It includes infiltration into a person’s existing accounts, using a person’s identity to open new accounts, or using personal information to obtain instrumental goods and services such as health care and public benefits (Harrell, 2019). Similar to financial fraud, the vast majority of identity theft victims do not have a preexisting personal relationship with the perpetrator. Yet unlike fraud, most incidents do not involve a direct exchange of information or payment. Rather, identifying information is taken and used without the victim’s knowledge or consent, such as through a data breach or malware attack.

Prior research demonstrates that victims experience severe monetary and nonmonetary consequences following financial victimization. Fraud victims report feeling embarrassed and ashamed, angry, stressed, and anxious, with some reporting depression and strained relationships with family and friends (Button et al., 2014; Financial Institution Regulatory Authority 2015). Sharp et al. (2003) found that maladaptive psychological and somatic symptoms increased post identity theft victimization. Longitudinal research has demonstrated that elder mistreatment, including financial exploitation, is associated with increased risks of poor mental and physical health outcomes (Acierno et al., 2017), hospitalization (Dong & Simon, 2013), and mortality (Lachs et al., 1998).

Negative financial, social, and emotional outcomes may be more prevalent and severe among older retired victims who lack employment opportunities to make up their losses or who are unable to navigate the process of resolving the incident with financial institutions and credit bureaus. Additionally, because older generations have relatively greater wealth than younger generations (Gale et al., 2020), they may experience higher levels of theft. Indeed, consumer fraud reports indicate that adults in their 80s experience 3–4 times higher median losses per scam ($1,600) than adults aged 20–49 (FTC, 2020).

Using data from 2012 and 2014 National Crime Victimization Survey (NCVS) Identity Theft Supplements (ITS), Burnes et al. (2020) showed that baby boomers were significantly more likely than millennials to be victims of identity theft. Results from the 2016 ITS show that older adults suffered an estimated $2.5 billion in financial losses (Harrell, 2019). In addition to direct losses, other costs include financial and legal troubles and ruined credit. These consequences may be more severe for older adults with physical or cognitive impairments that make it difficult to contact multiple credit bureaus and financial institutions to report identity misuse. Older adults also have lower knowledge of cybersecurity practices to safeguard their identities from continued misuse (Nicholson et al., 2019).

Several recent studies have examined the financial, psychological, and health consequences of identity theft among U.S. adults of all ages. The 2016 NCVS–ITS shows that 12% of victims experienced out-of-pocket costs, with average losses of $690 (Harrell, 2019). Reynolds (2020) found that unmarried victims and those with lower incomes and educational attainment were significantly more likely to experience out-of-pocket costs following identity theft, as were Hispanic/Latino respondents. Age was positively associated with out-of-pocket costs for incidents that involved misuse of bank account information. Reynolds (2020) also found that the risk of out-of-pocket costs differed by the type of identity theft, such that those who experienced misuse of credit card information were significantly more likely to be reimbursed than victims of bank account identity theft.

Using data from the 2012 NCVS–ITS, Randa and Reynes (2020) examined the predictors of emotional distress among all adults. Thirty-two percent of victims reported that the identity theft incident caused them moderate to severe distress. Older adults were significantly more likely to report distress, as were women and those with lower household incomes. The time spent resolving the incident with credit bureaus and financial institutions was also positively related to distress. Using the same data, Golladay and Holtfreter (2017) examined the emotional and physical consequences following identity theft victimization. Similarly, they found that older adults, minorities, and those who suffered higher losses reported an increasing number of emotional consequences—worry/anxiety, anger, depression, vulnerability, feeling unsafe, confused, violated, etc. There was also a negative association between emotional consequences and socioeconomic status, suggesting that those who are better off financially suffer less in the aftermath of victimization.

## Study Purpose

The current body of research suggests that identity theft victimization has a disproportionate negative impact on older adults and low-income people, but no studies have specifically examined the correlates of financial and psychological consequences among older victims. Using combined data on victims from the 2014 and 2016 NCVS–ITS, we examine how socioeconomic status, demographic characteristics, and other incident-related factors relate to the total amount stolen, out-of-pocket costs, and emotional distress among victims aged 65 and older, controlling for the type of identity theft experienced. Results offer insight into what groups are in greatest need of resources for emotional support and financial recovery, as well as greater identity protection.

## Method

### Sample

This study is restricted to respondents aged 65 and older who reported identity theft victimization occurring in the past 12 months in the 2014 and/or 2016 NCVS–ITS survey (*N* = 2,513). These cross-sectional ITS surveys were administered during 6-month periods in each of the years and are consistent in survey content and methodology. They were combined for additional statistical power and more robust estimates. The ITS is administered to respondents aged 16 and older at the end of their NCVS interview using computer-assisted personal interviewing or computer-assisted telephone interviewing. Respondents are asked whether they have experienced different types of misuse of identifying information during the prior year. Those who answer affirmatively are asked to think about the most recent incident and answer more detailed, incident-specific questions about the nature and consequences of the experience.

The current study focuses specifically on the aftermath of identity theft victimization (not attempted identity theft) where the older victim was not in a trust relationship with the perpetrator (financial abuse) and did not willingly provide their personal information to the perpetrator in response to a scam solicitation (fraud). Respondents are asked how long their information was misused before they discovered the identity theft. Those who selected “Not applicable, not actually misused” (i.e., *attempted* identity theft) were removed from the sample (*n* = 50, 1.9%). Victims who experienced identity theft resulting from a scam (i.e., stated that the incident occurred after they responded to a scam email/phone call [*n* = 45, 1.7%]) were also excluded. Although the vast majority of respondents did not know the identity of the perpetrator (93%), those who did and reported that it was a relative, caregiver, or someone working in the home, housemate, friend, or neighbor (*n* = 68, 2.5%) were excluded to avoid overlap with the definition of financial exploitation/abuse by a trusted individual.

The broader NCVS study uses a two-stage, stratified cluster sample design representing U.S. residents living in housing units or group quarters. The overall NCVS–ITS unit response rate was 66% in 2014 and 61% in 2016. Selection bias analysis found little or no bias to ITS estimates due to nonresponse (US Department of Justice, 2014, 2016). Data were weighted to reflect a nationally representative sample in regard to age, gender, and race/ethnicity and to compensate for survey nonresponse and aspects of the staged sampling design. Further details on NCVS–ITS methods and the survey instruments can be found at https://bjs.ojp.gov/.

### Dependent Variables

#### Total amount stolen

Respondents reported how much money (in dollars) identity thieves initially obtained in the incident, regardless of whether these losses were ultimately recovered or reimbursed. In nearly a third of the incidents, identity thieves did not obtain any money, but among those who had money stolen, median losses were $200.00 and mean losses were $1,111.04 (standard deviation [*SD*] = 4,877.70). Based on the distribution, values were recoded into four categories: $0 (reference category; 30% of total), $1–100 (25%), $101–500 (21%), and $501 and greater (17%). Approximately 7% (*n* = 201) of victims did not know how much money was stolen and were excluded from this analysis.

#### Out-of-pocket costs

Out-of-pocket costs are monetary losses that are not reimbursed or recovered following victimization. Because only 7% of older victims experienced out-of-pocket costs, this variable is treated as dichotomous where 0 = no loss and 1 = any loss. Of those who experienced an out-of-pocket cost (*n* = 161), the median loss amount is $200.00 with a mean of $1,453.47 (*SD* = 9,854.13). Those who did not know whether they suffered out-of-pocket costs (8%) were excluded from this analysis (*n* = 209).

#### Emotional distress

On a 4-point Likert scale, respondents were asked to rate how distressing the misuse of their personal information was to them. Responses included “not at all distressing,” “mildly distressing,” “moderately distressing,” and “severely distressing.” Following the convention used in prior studies (Golladay & Holtfreter, 2017; Randa & Reynes, 2020), the item was dichotomized such that those who rated their distress as moderate or severe were coded as “1” (34%).

### Independent Variables

#### Types of identity misuse

Because the likelihood of being reimbursed or having funds recovered varies based on the nature of identity theft, *types of identity misuse* were divided into five categories based on how the respondent answered the ITS victimization screening questions. The reference category is *existing credit card account*: “During the past 12 months, has someone used or attempted to use one or more of your existing credit cards without your permission?” (yes = 1). *Other existing accounts* include respondents who said yes to one or both of the following questions: “Has someone, without your permission, used or attempted to use your existing checking or savings account, including any debit or ATM cards?” (yes = 1), and/or “Has someone misused or attempted to misuse another type of existing account such as your telephone, cable, gas or electric accounts, online payment accounts like Paypal, insurance policies, entertainment account like iTunes, or something else?” Before answering the items on existing bank account and credit card identity theft, respondents were first asked if they owned either of these accounts. If not, that particular item was skipped. *New accounts* identity theft was measured using the question: “Has someone, without your permission, used or attempted to use your personal information to open any NEW accounts such as wireless telephone accounts, credit card accounts, loans, bank accounts, online payment accounts, or something else?” (yes = 1). The fourth category is instrumental identity theft that was measured using the following item: “Has someone used or attempted to use your personal information for some other fraudulent purpose, such as filing a fraudulent tax return, getting medical care, applying for a job or government benefits; giving your information to the police when they were charged with a crime or traffic violation, or something else?” (yes = 1). *Multiple types* of identity theft were defined as a single incident of information exposure (e.g., a stolen wallet) that results in the multiple types of identity theft as described in the categories above.

#### Socioeconomic indicators

Educational attainment was coded as 0 = less than high school, 1 = high school or GED equivalent, 2 = some college/Associate degree, and 3 = Bachelor’s degree or higher. Percent of federal poverty level (FPL) was an ordinal variable that measured a respondent’s household income as a percentage above, at, or below the FPL as determined by the U.S. Department of Health and Human Services. It is a more robust measure than simply using household income because it takes into account the household size. Harrell et al. (2014) provide additional information on how this measure was calculated.

#### Demographic characteristics

Age was coded continuously. Race was coded as 0 = White, non-Latino; 1 = Black/African American, non-Latino; 2 = Latino; 3 = other race/ethnicity, non-Latino. Sex was 1 = female. Marital status was 1 = married.

#### Incident-specific factors

Respondents were asked whether they experienced banking and/or credit problems following identity theft and if they were successful in clearing up the financial and credit issues associated with the misuse of their information. Those who said “yes” were coded as 1 = incident resolved. *Time to discovery* measured how much time passed between when the victim’s information was misused and when they discovered the misuse, where 0 = one day or less, 1 = more than a day but less than a week, 2 = at least a week, but less than 1 month, 3 = 1 month to less than 6 months, 4 = 6 months or more, and 5 = unknown. *Time to resolve* was measured continuously as the number of hours it took the victim to clear up any financial and/or credit problems associated with identity theft. Respondents were asked if the incident caused them to have significant problems with family members or friends, including getting into more arguments or fights, not feeling they could trust them as much, or not feeling as close to them as before (*Subsequent problems with family/friends*; 1 = yes). They were also asked if they experienced any credit-or banking-related problems as a result of identity theft, such as being turned down for a line of credit, a loan, or a checking account; having to pay a higher interest rate; or having checks bounce (*Subsequent financial and/or credit problems*; 1 = yes). They were also asked if they contacted a bank, credit card company, or other financial institution following the incident (*Contacted financial institution*; 1 = yes). This behavior may also affect whether the victim was able to recover all or a portion of their stolen funds or reverse unapproved charges. *Multiple ID theft incidents* measured whether the victim experienced other separate incidents of identity theft within the past 12 months (1 = yes), and *prior victimization* measured whether the respondent experienced identity theft victimization occurring prior to the past 12 months (1 = yes).

### Analysis

Population weights were applied in all analyses. Models were analyzed in SPSS 25 using complex samples procedures to account for the address-based sampling design of the NCVS. Using ordinal regression, the *total amount stolen* was regressed on demographic and socioeconomic victim characteristics (*N* = 2,307). The four levels of the dependent variable were $0 stolen (reference), $1–100, $101–500, and $501 or more. Additional independent variables included the type of identity theft (existing credit card = reference) and whether the victim contacted their financial institution to report the incident. Using logistic regression, *out-of-pocket costs* were regressed on the same demographic and socioeconomic characteristics, as well as the type of identity theft and whether the victim contacted their financial institution following the incident (*N* = 2,302).

In a final logistic regression, *emotional distress* was regressed on demographic and socioeconomic characteristics, type of identity theft, and other incident-specific factors (*N* = 2,160). These additional factors included banking and/or credit problems = 1, incident resolved = 1, time to discovery (ordinal), hours spent resolving incident (continuous), subsequent problems with friends/family = 1, subsequent financial and/or credit problems = 1, multiple identity theft incidents = 1, and prior identity theft victimization = 1. The sample size dropped due to missing responses on the additional independent variables.

## Results

### Sample Characteristics

Table 1 presents sample characteristics. Approximately half of the identity theft victims surveyed were female (51%) and 65% were married. The mean age was 72 years. Forty-four percent of victims had a bachelor’s degree or higher. The majority lived in a suburban environment (56%), followed by urban (28%) and rural (16%). Eighty-eight percent were White (non-Latino), 5% were Black, and 4% were Latino. In 2016, the federal poverty threshold for a two-person household was an annual household income of less than $16,000. Five percent of older identity theft victims in this sample were at or below 100% FPL (adjusted for their household size), whereas 36% were at or above 501% FPL.

**Table 1.** Weighted Sample Characteristics

Participant characteristics	Weighted *N*	Weighted %
Female	2,890,008	50.90
Married	3,674,241	64.80
*Urbanicity*		
Rural	908,903	16.00
Suburban	3,196,565	56.30
Urban	1,568,310	27.60
*Race*		
White (non-Latino)	4,992,850	88.00
Black	294,950	5.20
Latino	244,500	4.30
Asian/Indigenous/Pacific Islander/other	141,477	2.50
*Educational attainment*		
Less than high school diploma	391,801	6.90
High school graduate	1,167,466	20.60
Some college/associate degree	1,580,896	27.90
College degree or more	2,533,614	44.70
*Income as percent of federal poverty level*		
0–100%	279,629	4.90
101–150%	306,731	5.40
151–200%	446,634	7.90
201–300%	1,109,535	19.60
301–400%	836,526	14.70
401–500%	643,530	11.30
501% or higher	2,051,194	36.20
*Type of identity theft*		
Existing credit card identity theft	1,355,246	23.90
Multiple types of identity theft	3,686,820	65.00
Other existing account identity theft	132,850	2.30
New account identity theft	98,717	1.70
Instrumental identity theft	400,145	7.10
*Amount of time info used prior to discovery*		
One day or less (1–24 hours)	2,552,787	45.00
More than a day, but less than a week (25 h–6 days)	1,246,356	22.00
At least a week, but less than one month (7–30 days)	804,362	14.20
One month to less than six months	517,243	9.10
Six months or more	85,347	1.50
Unknown	467,683	8.20
Incident was resolved	5,269,221	92.90
Subsequent financial and/or credit problems	153,707	2.70
Subsequent family/friend relationship problems	63,942	1.10
Multiple identity theft incidents in 12 months	1,344,624	23.70
Prior identity theft (more than 12 months ago)	1,257,943	22.20

### Total Amount Stolen

Few victim characteristics were associated with the total amount of money stolen (Model 1, Table 2). However, older Black victims of identity theft were more likely to have higher dollar amounts stolen than older White victims (odds ratio [OR] = 1.50, 95% confidence interval [CI] = 1.08–2.07, *p* = .016). Victim age, sex, educational attainment, marital status, area of residence, and poverty level were not associated with the amount stolen. Type of identity theft reported was significant such that experiencing multiple types of identity theft was related to increasing amounts of money stolen relative to existing credit card identity theft (OR = 1.59, 95% CI = 1.11–2.27, *p* = .012). Amount stolen was *negatively* associated with contacting a financial institution (OR = 0.41, 95% CI = 0.28–0.61, *p* < .001), meaning that having less money stolen decreases the odds of contacting a financial institution by nearly 60%.

**Table 2.** Factors Associated With Increasing Amounts of Money Stolen and Out-of-Pocket Costs Following Identity Theft

Independent variables	Model 1: Total amount stolen (*N* = 2,307)				Model 2: Out-of-pocket costs (*N* = 2,302)			
	Odds ratio	95% CI		p	Odds ratio	95% CI		p
		Lower	Upper			Lower	Upper	
Age	1.01	0.99	1.02	.355	1.01	0.98	1.05	.342
Female	0.96	0.82	1.13	.618	0.75	0.51	1.10	.138
Married	0.88	0.73	1.05	.160	0.54	0.34	0.84	.007
*Urbanicity*								
Urban (reference)	—	—	—	—	—	—	—	—
Rural	1.04	0.81	1.34	.744	1.60	0.90	2.84	.109
Suburban	0.96	0.80	1.16	.667	0.89	0.57	1.40	.615
*Race/ethnicity*								
White non-Latino (Reference)	—	—	—	—	—	—	—	—
Black	1.50	1.08	2.07	.016	1.86	0.90	3.83	.093
Latino	1.18	0.69	2.04	.540	0.56	0.20	1.56	.264
Asian/Indigenous/Pacific Islander/other	1.06	0.66	1.72	.802	3.60	1.69	7.67	.001
*Educational attainment*								
Less than high school diploma (Reference)	—	—	—	—	—	—	—	—
High school graduate	0.94	0.66	1.35	.748	0.98	0.43	2.22	.957
Some college/associate degree	1.14	0.78	1.64	.498	0.93	0.44	1.98	.855
College degree or more	1.07	0.75	1.53	.713	1.00	0.45	2.19	.993
*Income as percent of federal poverty level* ^a^								
0–100%	1.16	0.79	1.71	.454	4.93	2.50	9.73	<.001
101–150%	0.92	0.62	1.36	.672	1.84	0.79	4.28	.155
151–200%	0.92	0.65	1.30	.625	1.03	0.38	2.76	.956
201–300%	0.90	0.67	1.20	.465	1.68	0.93	3.03	.087
301–400%	1.03	0.75	1.40	.872	1.33	0.72	2.45	.358
401–500%	1.20	0.92	1.57	.178	1.53	0.79	2.96	.201
501% or greater (Reference)	—	—	—	—	—	—	—	—
*Type of identity theft*								
Existing credit card identity theft (Reference)	—	—	—	—	—	—	—	—
Multiple types of identity theft	1.59	1.11	2.27	.012	1.02	0.52	2.01	.950
Other existing account identity theft	1.06	0.88	1.27	.560	0.54	0.35	0.83	.005
New account identity theft	0.49	0.22	1.07	.075	0.52	0.16	1.67	.269
Instrumental identity theft	0.36	0.09	1.45	.150	1.80	0.31	10.52	.512
Contacted financial institution	0.41	0.28	0.61	<.001	0.96	0.46	2.00	.911

^a^Incorporates household size.

### Out-of-Pocket Costs

As given in Model 2, Table 2, more socioeconomic and demographic characteristics were associated with out-of-pocket costs, which are financial losses that are not reimbursed by financial institutions or recovered by victims. Older victims who identified as single (divorced, widowed, separated, never married) were significantly less likely than older married victims to have out-of-pocket costs (OR = 0.54, 95% CI = 0.34–0.84, *p* = .007). In addition to experiencing significantly higher amounts stolen, older Black victims showed a trend (*p* < .1) toward being more likely to experience out-of-pocket costs (OR = 1.86, 95% CI = 0.90–2.84, *p* = .09). Victims who identified their race as “other,” which includes the categories of Asian, Pacific Islander, and Indigenous, were significantly more likely to suffer out-of-pocket costs than older White victims (OR = 3.60, 95% CI = 1.69–7.67, *p* = .001). Those living at or below the FPL (0–100% FPL) were significantly more likely to experience out-of-pocket costs relative to those living at 501% or more FPL (OR = 4.93, 95% CI = 2.50–9.73, *p* < .001). Relative to those who reported existing credit card identity theft, those who reported other existing account identity theft were significantly less likely to suffer out-of-pocket costs (OR = 0.54, 95% CI = 0.35–0.83, *p* = .005), suggesting that this type of identity theft is less likely to involve financial losses. Victim age, sex, educational attainment, and urbanicity were not significantly associated with out-of-pocket costs. Unlike the total amount stolen, whether the victim contacted their financial institution was not significant for out-of-pocket costs.

### Emotional Distress

Table 3 presents the results of emotional distress regressed on victim demographic and socioeconomic characteristics, along with incident-related factors that may affect psychological outcomes following victimization. Female victims were 40% more likely to report distress than male victims (OR = 1.40, 95% CI = 1.13–1.74, *p* = .002). Relative to older White victims, older Black victims were 76% more likely to experience emotional distress (OR = 1.76, 95% CI = 1.14–2.70, *p* = .010), and those who identified their race/ethnicity as “other” were 46% *less likely* to report distress (OR = 0.54, 95% CI = 0.30–0.96, *p = .*034).

**Table 3.** Factors Associated With Emotional Distress Following Identity Theft (*N* = 2,160)

Independent variables	Odds ratio	95% CI		p
		Lower	Upper	
Age	1.00	0.98	1.03	.705
Female	1.40	1.13	1.74	.002
Married	1.15	0.91	1.46	.237
*Urbanicity*				
Urban (Reference)	—	—	—	—
Rural	1.10	0.79	1.51	.575
Suburban	1.08	0.85	1.38	.512
*Race/ethnicity*				
White non-Latino (Reference)	—	—	—	—
Black	1.76	1.14	2.70	.010
Latino	0.79	0.42	1.48	.452
Asian/Indigenous/Pacific Islander/other	0.54	0.30	0.96	.034
*Educational attainment*				
Less than high school diploma (Reference)	—	—	—	—
High school graduate	1.43	0.88	2.31	.145
Some college/associate degree	1.45	0.91	2.31	.119
College degree or more	1.36	0.83	2.21	.223
*Income as percent of federal poverty level* ^a^				
0–100%	0.71	0.40	1.25	.236
101–150%	1.35	0.78	2.36	.285
151–200%	1.21	0.79	1.84	.377
201–300%	1.06	0.76	1.47	.731
301–400%	1.11	0.76	1.61	.588
401–500%	1.23	0.86	1.75	.254
501% or greater (Reference)	—	—	—	—
*Type of identity theft*				
Existing credit card identity theft (Reference)	—	—	—	—
Multiple types of identity theft	0.83	0.51	1.36	.458
Other existing account identity theft	0.69	0.53	0.89	.004
New account identity theft	1.84	0.91	3.71	.089
Instrumental identity theft	1.45	0.55	3.82	.445
*Total amount stolen*				
$0 (Reference)	—	—	—	—
$1–$100	1.10	0.74	1.39	.929
$101–$500	1.38	1.02	1.87	.038
$501 or more	2.46	1.76	3.44	<.001
*Length of time information was misused prior to discovery*				
One day or less (Reference)	—	—	—	—
More than a day, but less than a week	1.32	0.99	1.78	.062
At least a week, but less than 1 month	1.58	1.18	2.14	.003
One month to less than 6 months	2.21	1.53	3.18	<.001
Six months or more	1.21	0.55	2.67	.640
Unknown	1.14	0.79	1.66	.478
*Other incident characteristics*				
Experienced out-of-pocket costs	1.87	1.11	3.14	.018
Incident was resolved	1.10	0.73	1.67	.648
Number of hours spent resolving the incident	1.03	1.02	1.05	<.001
Subsequent financial and/or credit problems	1.94	1.02	3.71	.045
Subsequent problems with friends/family	11.59	2.31	58.16	.003
Multiple incidents within the past 12 months	1.41	1.10	1.81	.007
Prior identity theft victimization	0.92	0.70	1.22	.574

^a^Incorporates household size.

Victims who suffered out-of-pocket costs were 87% more likely to report emotional distress relative to those with no out-of-pocket costs (OR = 1.87, 95% CI = 1.11–3.14, *p* = .018). Even after controlling for out-of-pocket costs, those who had between $101 and $500 stolen were 38% more likely to feel distressed (OR = 1.38, 95% CI = 1.02–1.87, *p* = .038), and those who had $501 or more stolen were two and a half times as likely to feel distressed (OR = 2.46, 95% CI = 1.76–3.44, *p* < .001) compared to those who had no money taken. Other existing account identity theft was negatively associated with experiencing distress (OR = 0.69, 95% CI = 0.53–0.89, *p* = .004). Relative to those who discovered their identity had been misused within the same day of the theft, those who discovered it a week to 1 month later were 58% more likely to feel distressed (OR = 1.58, 95% CI = 1.18–2.14, *p* = .003), and those who did not discover the incident until 1 month to 6 months later were more than twice as likely to feel distressed (OR = 2.21, 95% CI = 1.53–3.18, *p* < .001), although discovering the incident more than 6 months later had no effect. Experiencing multiple incidents of identity theft within the same year was also significantly associated with distress (OR = 1.41, 95% CI = 1.10–1.81, *p* = .007). Older victims who reported that identity theft led to subsequent financial and credit problems were 94% more likely to experience emotional distress (OR = 1.94, 95% CI = 1.02–3.71, *p* = .045), and those who stated that the incident negatively affected their relationships with friends and/or family members were 11 times more likely to report moderate to severe emotional distress (OR = 11.59, 95% CI = 2.31–58.16, *p* = .003).

## Discussion

This is the first study to examine the financial and psychological outcomes of identity theft among older adult victims. Although only 7% of older victims experience out-of-pocket costs associated with identity theft, 34% describe the experience as moderately to severely distressing, indicating that the harm resulting from personal information misuse extends beyond direct financial losses.

Incident-specific factors are important contributors to distress. The more money that is stolen from the victim during the incident, the greater the odds of emotional distress, regardless of whether losses are recovered or reimbursed. Also, the longer information is misused before the crime is discovered, the more subsequent financial and credit problems, and the more hours spent resolving the incident, the greater the likelihood of distress. Our findings reflect results from a smaller survey of a few hundred adult victims that found that the magnitude of financial loss, the duration of misuse of personal information, and the amount of time spent resolving the effects of the crime are all factors that increase perceived distress (Li et al., 2019).

Beyond incident-specific characteristics, we find that older Black victims and older female victims are significantly more likely to report emotional distress, controlling for other demographic and socioeconomic characteristics. Prior work using the ITS shows that minorities experience higher levels of distress than Caucasian individuals (Golladay & Holtfreter, 2017), and Burnes et al. (2020) found that Black respondents were 58% more likely to report instrumental identity theft relative to other race and ethnic groups. This subtype of identity theft may be particularly stressful for victims because it involves using the victim’s personal identity to obtain benefits and services that the victim is entitled to, such as health care, tax refunds, and enrollment in government programs. The higher prevalence of instrumental identity theft in Black communities may help account for their higher levels of distress, although this type of identity theft was not significantly associated with distress in the current models.

Older Black victims were also more likely to have increasing amounts of money stolen relative to older White victims, although there were no statistically significant differences in out-of-pocket costs. Rather, those who are Asian, Pacific Islander, Indigenous, mixed race, or other race/ethnicity were more likely to suffer out-of-pocket costs. Controlling for out-of-pocket costs, this group was counterintuitively *less* likely to report emotional distress following the incident. More research is needed to better understand the relationship between identity theft and distress among older adults who belong to these minority groups.

We find that the poorest older Americans are more likely to suffer out-of-pocket costs. Specifically, relative to the wealthiest victims aged 65 and older, those who live at or below the FPL are nearly 5 times as likely to bear a financial burden following the incident, even after accounting for the type of identity theft and whether the victim contacted their financial institution. Consistent with findings from the general U.S. adult population (Copes et al., 2010; Reynolds, 2020), our results illustrate the importance of social and economic capital in addressing identity theft incidents. To resolve identity theft, the FTC recommends that victims contact their financial institutions or the company involved in the incident, change their passwords, request that money be reimbursed or charges reversed, contact all three credit bureaus to place fraud alerts, and report the incident to authorities. Depending on the severity of the incident, victims may also need to place a freeze on their credit, write to credit bureaus to request corrections to their credit reports, close unauthorized new accounts, write to debt collectors explaining the situation, report to the Social Security Administration, and replace government-issued IDs. These tasks can place a tremendous burden on low-income older adults, many of whom lack access to broadband internet, supportive ties who can advocate on their behalf, or the knowledge and wherewithal to negotiate with powerful financial institutions. Research is needed to determine whether wealthy and/or White older adults are treated differently by their financial institutions when they report identity theft, and whether they are more likely to have account safeguards in place or a client/customer status that helps keep their information safe.

This is the first study to show the negative impact of identity theft on social relationships after controlling for other victim and incident-level characteristics. Maintaining strong positive social and emotional relationships is critical for health and well-being in later life (Cho et al., 2015; Litwin & Shiovitz-Ezra, 2011). Findings here illustrate that victims who reported that identity theft caused significant problems with family members or friends were 12 times as likely to experience emotional distress, suggesting that identity theft can have severe ramifications for older adults’ well-being. Qualitative research is needed to understand how identity theft victimization leads to relationship discord. One possibility is that family members blame the older victim for the incident, assuming that they did not keep their personal information secure or that they waited too long to take action. Victim blaming is common in fraud and is likely a driver of low rates of reporting (Cross, 2015; Cross et al., 2016). Future studies are needed to understand the role that family and friends play in helping victims recover from identity theft. Family participation in working to protect an older adult from identity crimes, such as providing account oversight and coaching on cybersecurity practices, may be a critical factor in keeping them safe against future victimization.

### Implications and Future Research

Findings suggest that limiting the extent of losses and reducing the length of time information is misused prior to detection may reduce the emotional toll of identity theft. Older adults in particular should increase surveillance of their identifying information by using identity protection software, two-step authentication features, signing up for credit alerts, and applying low spending limits on credit cards. Other personal protection behaviors, such as routinely changing passwords, making passwords complicated and varying them for each account, monitoring financial transactions, and locking up or shredding documents, are also important for preventing identity theft. Future research should examine the impact of these identity protection behaviors specifically among older adults. Moreover, this study excluded many older adults who experienced *attempted* identity theft. Using NCVS–ITS data, additional research may explore how these individuals differ from victims, particularly in regards to their identity protection behaviors.

Given that the length of identity misuse is strongly related to emotional distress, financial institutions should act swiftly to stop suspicious transactions before charges can escalate, and organizations should not delay in informing their customers, employees, and law enforcement of data breaches that involve personal or payment information. Unfortunately, Lacey and Cuganesan (2004) report that a minority of organizations report possible data breaches to law enforcement agencies, indicating that consumers also fail to learn about potential information exposure.

Like identity theft, very little research has been done to examine the outcomes of fraud victimization on older adults. To that end, the Bureau of Justice Statistics recently released a new fraud supplement that assesses the prevalence of different types of fraud. The questionnaire includes information on the amount lost and the emotional impact on victims. Although the amount varied by scam type, victims lost an average of $700 per incident and 53% reported socioemotional problems as a consequence of victimization (Morgan, 2021). Future research should compare how the outcomes of fraud victimization compare to the outcomes of identity theft, and whether Black and female victims also experience higher levels of emotional distress.

Some research has explored how identity theft might affect consumers’ trust in the marketplace, particularly their confidence and willingness to engage in online transactions (Chakraborty et al., 2016; Roberts et al., 2013). Avoiding the transfer of personal information online is near impossible in today’s society, as most companies and government agencies rely on the internet to do business with consumers. Future research should examine how identity theft victimization affects older consumers’ trust in government agencies and other institutions, and whether it affects online shopping and sharing of personal information in online environments.

The coronavirus pandemic has created new risks of identity theft as many older adults have turned to the internet to meet their shopping, banking, and even health care consultation needs. While the NCVS–ITS data used in this study were collected prior to the pandemic, it should be noted that identity theft was prevalent following the steep rise in joblessness that disproportionately affected low-income and minority workers. International criminals filed for U.S. unemployment benefits using the stolen identities of American citizens, siphoning off approximately $36 billion from the program, or 10% of all funds expended for unemployment benefits under the CARES Act (Office of the Inspector General, 2020). It is unknown how these crimes affected older adults in particular, and whether they have influenced older adults’ confidence in exchanging personal information with the government.

### Limitations

Although the ITS is one of the most comprehensive sources of data on identity theft, the survey excludes individuals with severe cognitive impairment and those who live in institutional settings (e.g., psychiatric care, long-term care, nursing homes). The impacts of identity theft on these vulnerable older adults are not known, although victim research on fraud indicates that cognitive decline and dementia are correlates of increased risk (Boyle et al., 2019).

Unfortunately, the ITS does not include measures of whether older adults may be experiencing cognitive decline or other mental or physical health conditions that could affect distress and the ability to recover losses. Moreover, the ITS uses a 1-year reference period and it may be difficult for older victims with cognitive impairment to accurately remember details of the incident and how they felt about it. Identity theft is an unusual crime in that the consequences, such as diminished credit scores or unexplained credit card charges, may be overlooked by some victims and therefore underreported.

Although the survey has relatively high response rates and no strong evidence of bias, it is possible that older adults who refuse to participate in the NCVS or the ITS may be more reluctant to provide personal information in a survey because they have experienced identity theft previously. This would mean that more victims in the nonresponse group are not represented in the data.

Emotional distress was measured as a single item and was recoded from four levels into a dichotomous variable. Although this has been the convention used in prior studies (Golladay & Holtfreter, 2017; Randa & Reynes, 2020), a binary treatment reduces information and may conceal nonlinear relationships between distress and other variables. To test for differences in effects, we performed a post hoc ordinal regression. Emotional distress (four levels) was regressed on the same independent variables. Only two substantive differences emerged: Victims of other race/ethnic backgrounds were no longer significantly less likely to experience distress relative to non-Hispanic White victims (*p* = .318) and experiencing more than one separate incident of identity theft within the past year was only marginally associated with distress (*p* = .066). Future research should consider using a more comprehensive, multi item measure of distress.

### Conclusion

Findings from this study largely align with studies that examine the impact of identity theft victimization on adults of all ages, although older adults may present additional vulnerabilities, such as cognitive decline and isolation, which could increase their risk of serious outcomes. New programs and services are needed to help older victims recover, with a particular focus on low-income people and those who lack the ability to advocate for themselves. Advocates may assist older victims with contacting multiple financial institutions and credit bureaus, filing complaints, and freezing their credit. Additional services might include victim support groups and other psychological resources, as well as information for family caregivers on how to support older victims. Future research should assess whether cybersecurity training can help older adults secure their identity information and reduce their risk of future identity crimes.
